# Evaluating Ice-Temperature Storage Efficacy on Volatile Compounds in Blue Honeysuckle (*Lonicera caerulea* L.) by Combining GC-IMS and GC-MS

**DOI:** 10.3390/foods14071205

**Published:** 2025-03-29

**Authors:** Tianbo Li, Xiaoyu Jia, Jiangkuo Li, Peng Zhang, Dong Qin, Di Wu, Tong Chen, Junwei Huo

**Affiliations:** 1 College of Horticulture and Landscape Architecture, Northeast Agricultural University, Harbin 150006, China; litianbo20@163.com (T.L.); dongq9876@126.com (D.Q.); 2 Institute of Agricultural Products Preservation and Processing Technology, Tianjin Academy of Agricultural Sciences, Tianjin 300384, China; jiaxiaoyu331@outlook.com (X.J.); lijkuo@sina.com (J.L.); zhangpeng811202@163.com (P.Z.); 3 Tianjin Key Laboratory of Postharvest Physiology and Storage of Agricultural Products, National Engineering and Technology Research Center for Preservation of Agricultural Products, Tianjin 300384, China; 4 Zhejiang University Zhongyuan Institute, Zhengzhou 450001, China; di_wu@zju.edu.cn; 5 State Key Laboratory of Plant Diversity and Specialty Crops, Institute of Botany, Chinese Academy of Sciences, Beijing 100093, China

**Keywords:** blue honeysuckle, ice temperature storage, GC-IMS, GC-MS, volatile compounds, TEM, small berry

## Abstract

This study evaluated the efficacy of ice-temperature storage (−1 °C) in preserving volatile compounds (VOCs) in blue honeysuckle (*Lonicera caerulea* L.) as compared to conventional low-temperature (4 °C) and freezing (−3 °C) storage for 84 d with a 14 d interval. As a flavor-rich berry highly susceptible to postharvest VOC loss, VOC contents and ultrastructural variations were systematically analyzed by coupling gas chromatography–ion mobility spectrometry (GC-IMS), gas chromatography–mass spectrometry (GC-MS), and transmission electron microscopy (TEM). GC-IMS and GC-MS detected 25 and 62 VOCs, respectively, with ice-temperature storage demonstrating well maintaining VOC varieties and relative concentrations. Moreover, TEM analysis further revealed that ice-temperature storage maintained normal cellular ultrastructure integrity, particularly in cell wall organization and organellar morphology. These results conclusively establish ice-temperature storage as the optimal method for preserving both biochemical composition and cytological architecture in blue honeysuckle, thereby providing a scientific foundation for optimizing postharvest protocols and advancing cold-chain technologies for perishable berry fruits.

## 1. Introduction

Blue honeysuckle (*Lonicera caerulea* L.), a perennial shrub in the Caprifoliaceae family (Juss.), is commonly referred to as honeyberry, sweetberry honeysuckle, or haskap berry [1,2]. This species demonstrates broad biogeographical distribution across temperate regions of the northern hemisphere [3,4]. In its native Chinese habitat, the plant predominantly thrives in northeastern provinces where systematic domestication efforts have yielded numerous improved cultivars. The phytochemical composition of blue honeysuckle berries contributes to their recognized nutraceutical value, with research confirming various bioactive compounds associated with human health benefits [5,6,7,8]. Notably, its organoleptic profile presents a complex flavor matrix characterized by a distinctive bitter undertone that has attracted specific research attention. As a soft-skinned fruit, postharvest preservation poses significant technological challenges, particularly regarding flavor stability and maintenance of characteristic sensory attributes during storage [9,10,11,12]. As a critical quality determinant for consumer acceptance, fruit flavor has prompted extensive investigation into volatile compounds (VOCs) in blue honeysuckle. Xia et al. (2023) [13] employed two-dimensional gas chromatography–olfactometry–mass spectrometry (GC × GC-O-MS) to characterize 68 VOCs across eight cultivars, establishing sensory descriptors encompassing fruity, floral, herbaceous, saccharine, and acidic attributes. Their analysis identified six key odor-active constituents: linalool, hexanal, eucalyptol, octanal, nonanal, and ethyl 2-methylbutyrate. A follow-up study performed by Kupska et al. (2014) [14] carried out longitudinal analyses (2009–2012) on 11 cultivars using GC × GC coupled with time-of-flight mass spectrometry (GC × GC-TOFMS), revealing 44 terpenoid derivatives with eucalyptol, linalool, (+)-limonene, and (+)-α-terpineol demonstrating high abundance.

Ice-temperature (IT) storage, a controlled postharvest protocol maintaining subzero temperatures (typically −0.5 °C to −2 °C) while preventing tissue crystallization, has emerged as an advanced preservation strategy in horticultural sciences and food engineering [15,16,17]. Its efficacy was systematically demonstrated in stone fruit preservation by Zhao et al. (2019) [18], who observed that IT storage extended sweet cherry shelf life by 35% compared to conventional refrigeration. Their biochemical analyses revealed significant maintenance of nutraceutical components, including anthocyanins (82.3 ± 1.7%), reducing sugars (94.5 ± 0.9%), ascorbic acid (76.4 ± 2.1%), and phenolic acids (89.2 ± 1.4%), alongside mitigation of membrane degradation biomarkers—specifically, membrane permeability decreased by 41.2% and malondialdehyde accumulation was suppressed by 57.8% relative to control groups. By contrast, Liu et al. (2019) [19] conducted volatile organic compound (VOC) profiling on *Prunus armeniaca* ‘Shushanggan’ under IT conditions. Through targeted metabolomics, they quantified 10 aroma determinants, notably hexanal and γ-decalactone, showing 68% and 73% retention, respectively, after 28-day storage. Comparative analyses revealed IT storage outperformed both ambient (25 °C) and standard cold storage (4 °C) in preserving characteristic aroma profiles.

Gas chromatography–ion mobility spectrometry (GC-IMS) represents a cutting-edge analytical technique that integrates orthogonal separation principles through gas-phase electrophoretic mobility differentiation. This dual-dimensional detection system offers distinct advantages, including rapid analysis (<30 min/sample), parts-per-trillion (ppt) level sensitivity, and non-destructive characterization, without requiring sample pretreatment [20,21,22]. Its unique capability to generate three-dimensional topographic fingerprints (retention time × drift time × signal intensity) enables real-time visual metabolomic profiling, particularly advantageous for high-throughput screening applications involving complex volatile matrices. While substantial progress has been made in food science applications—exemplified by its implementation in soy-based fermentation matrices, extra virgin olive oil authentication, and enological aroma evolution tracking. However, its potential in postharvest biology of fresh produces remains underexplored. Current literature primarily documents its use in plant variety discrimination through volatile signatures [23,24]. Notably, its implementation in perishable produce preservation, particularly for monitoring dynamic flavor alterations during fruit storage, constitutes a novel research frontier.

This study establishes a multimodal analytical framework to elucidate the impacts of IT storage on blue honeysuckle flavor by integrating volatile profiling through orthogonal detection methodologies (GC-IMS and GC-MS) with quality attribute quantification and ultrastructural observations. This methodological framework aims to elucidate temperature-modulated VOC production patterns during postharvest storage, thereby providing empirical evidence to optimize cold chain protocols for flavor preservation in blue honeysuckle berries. Moreover, flavor stability was correlated with ultrastructural adaptations under IT conditions, providing mechanistic insights into cold stress response pathways in perishable berries. Practically, these findings may enable precision cold chain optimization for blue honeysuckle. Furthermore, the established GC-IMS/GC-MS synergy protocol offers a transferable model for postharvest flavoromics research across small fruit taxa.

## 2. Materials and Methods

### 2.1. Plant Materials and Storage Conditions

The *Lonicera caerulea* var. ‘Lanjingling’ berries were harvested from standardized cultivation plots at Northeast Agricultural University’s Horticultural Research Station on 19 June 2023. Fruits at commercial maturity were manually collected during the morning circadian window (06:00–08:00 CST) to minimize metabolic variation. Those free from mechanical damage and diseases were harvested and gently kept in plastic containers. Each customized container, designed to reduce injury caused by bumps during transportation, can hold approximately 2 kg of blue honeysuckles. Each unit load underwent precooling (4.0 ± 0.2 °C) for 24 h, then was transported to the National Agricultural Products Freshness Engineering and Technology Research Center (Tianjin, China) through a 2 °C cold chain transportation system.

The fruits were allocated into three cryo-preservation cohorts with thermal stratification upon arrival, namely conventional refrigeration (LT: 4.0 ± 0.3 °C), ice-temperature storage (IT: −1.0 ± 0.3 °C) and frozen temperature storage (FT: −3.0 ± 0.3 °C). A stratified random sampling protocol was executed at 14-day intervals, with one container randomly selected from each treatment. The experiments were performed for at least three biological replicates.

### 2.2. Determination of Freezing Point

The freezing point was determined as described by Fan et al. (2018) [25], with minor modifications. Twenty blue honeysuckles were randomly selected and wrapped with aluminum foil, with a temperature logger probe inserted into the fruits kept at −20 °C. The temperature at the fruit core was automatically recorded every 20 s until the fruits were completely frozen. A freezing curve was generated based on the collected data, while the freezing point was determined from the curve as follows: during the freezing process, the temperature profile exhibits a tendency to first drop below 0 °C, then rise for a while, before dropping again. The highest temperature point to which it rose after reaching 0 °C was considered as the freezing point.

### 2.3. Determination of VOCs by GC-IMS

The determination of VOCs by GC-IMS was performed according to the method of Zhou et al. (2023) [24], with minor modifications. A total of 2 g homogenate from fresh blue honeysuckle fruit was incubated in a 20 mL head-space bottle for 20 min at 50 °C, with the incubator set at 500 rpm. The sample was injected into the analyzer using an auto sampler (PAL RSI, PAL SYSTEM, Basel, Switzerland), with the syringe temperature set at 85 °C and an injection volume of 500 μL.

GC conditions: The temperature of the chromatographic column MXT-5 (15 m × 0.53 mm, 1.0 μm) was set to 60 °C, and the analysis lasted for 20 min. Initially, the carrier gas flow rate was 2 mL/min, held for 2 min, then increased linearly to 10 mL/min, further held for another 8 min, and finally increased linearly to 100 mL/min, and maintained for 2 min. IMS conditions: The drift tube length was 5.3 cm and set at 45 °C, with a gas flow rate of 75 mL/min. High-purity nitrogen (purity ≥ 99.999%) was used for all experiments. The measurements were conducted on days 0, 42, and 84.

Qualitative analysis: LAV 0.4.03 software (included in the instrument) was employed for analysis, with C4–C9 as the external standard substance, to calculate the retention index (RI) of volatile substances. The qualitative analysis was performed by comparing the retention index and relative migration time with NIST and IMS databases.

$$RI={RI}_{\left(Z\right)}+\frac{\left[{RI}_{\left(Z+1\right)}\right.-\left.{RI}_{\left(Z\right)}\right]\times \left[{log}_{t}^{R_{\left(X\right)}}\right]-{log}_{t}^{R_{\left(Z\right)}}}{\left[{log}_{t}^{R_{\left(Z+1\right)}}\right.-\left.{log}_{t}^{R_{\left(Z\right)}}\right]}$$

In the formula, *Z* and *Z* + 1, respectively, represent the number of carbon atoms contained in the normal ketone before and after the outflow of the target compound (X); RI_(*Z*+1)_ is the retention index of normal ketones containing the same number of carbon atoms after the target compound flows out; RI_(*Z*)_ is the retention index of normal ketones containing the same number of carbon atoms before the compound flows out; R_(*X*)_ is the retention time of the target compound; R_(*Z*)_ is the retention time of the normal ketone containing the same number of carbon atoms before the target compound flows out; R_(*Z*+1)_ is the retention time of a normal ketone containing the same number of carbon atoms after the target compound flows out.

### 2.4. Determination of VOCs by GC-MS

The determination of VOCs by GC-MS was carried out according to Xia et al. (2023) [13] with minor modifications. Volatile components were extracted using solid-phase microextraction (SPME) as follows: the blue honeysuckle was pulped using a wall-breaker and centrifuged at 10,000 rpm. for 20 min. An 8 mL aliquot of the upper pomace-free layer was aspirated and added to the headspace flask, followed by the addition of 30 μL of 2-methyl-3-heptanone (9.75 ng/μL), 2.5 g of sodium chloride (to increase extraction efficiency by salting out), and a magnetic stirring bar. 2-methyl-3-heptanone was chosen as the internal standard solution. The headspace vials were incubated at 45 °C for 20 min, and the volatiles were extracted using a DVB/CAR/PDMS fiber (50/30 μm, Supelco, PA, USA) at 45 °C for 40 min under constant stirring. After extraction, the extraction needle was promptly inserted into the GC injection hole and released at 250 °C for 5 min. Subsequently, the detection was carried out using Agilent GC-MS 8890A-5977B (Agilent Technologies Inc., Sanra Clara, CA, USA) with the initial chromatographic column temperature set at 40 °C and held for 3 min, followed by a temperature ramp-up to 230 °C at a rate of 5 °C/min. The electron ionization energy of the mass selective detector was set to 70 eV, with the ion source temperature set to 230 °C, the quadrupole temperature set to 150 °C, and the scanning range set to 35–550. High-purity helium gas was used as the carrier gas. The measurements were performed on days 0, 42, and 84.

### 2.5. Determination of Quality Attributes

The total soluble solids (TSS) content was determined using a pocket refractometer (PAL-1, ATAGO, Tokyo Japan). Approximately 40 g of blue honeysuckle was homogenized with a blender and filtered. The results were expressed as °Brix [26]. Titratable acidity (TA) was measured using an automatic potentiometric titrator (916 Ti-Touch, Metrohm, Herisau, Switzerland). A total of 20 g (accurate to 0.001 g) fruit homogenate was diluted to 250 mL with distilled water, heated in a water bath at 80 °C for 30 min, cooled to room temperature and filtered. A 20 mL aliquot of the filtrate was combined with 40 mL of distilled water and titrated with 0.05 mol/L of sodium hydroxide solution. The results were expressed as a percentage. Total anthocyanin (TAN) content was determined using the pH-differential method and expressed as mg/100 g [27]. Ascorbic acid (AsA) content was measured using a molybdenum blue colorimetric method and expressed as mg/100 g.

### 2.6. TEM Analysis

Blue honeysuckle fruits from three treatments were randomly selected for TEM observations on days 0 and 84 of storage. A 3 mm wide piece was cut horizontally from each fruit and immediately immersed in 4% glutaraldehyde fixative at 4 °C.

Procedures for sample preparation and TEM assay: The tissue was prepared into 1 mm^3^ small pieces with a surgical knife. The 1 mm^3^ tissue blocks were transferred into an EP tube with fresh TEM fixative for further fixation, followed by vacuum extraction until the samples sink to the bottom. The samples were fixed for 2 h at room temperature and then fixed at 4 °C before further experiments. The tissues were rinsed with 0.1 M PBS (pH 7.4) for 3 times, 15 min each, then post fixed with 1% OsO4 in 0.1 M PBS (pH 7.4) for 7 h in dark at room temperature. After removing OsO4, the tissues were rinsed in 0.1 M PBS (pH 7.4) for 3 times, 15 min each, then dehydrated at room temperature as follows: 30% ethanol for 1 h; 50% ethanol for 1 h; 70% ethanol for 1 h; 80% ethanol for 1 h; 95% ethanol for 1 h; 100% ethanol for 1 h; 100% ethanol for 1 h; ethanol: Acetone = 3:1 for 0.5 h; ethanol: Acetone = 1:1 for 0.5 h; ethanol: Acetone = 1:3 for 0.5 h; Pure acetone for 1 h. Resin penetration and embedding were conducted as follows: Acetone: Epon 812 = 3:1 for 2–4 h at 37 °C; Acetone: Epon 812 = 1:1 overnight at 37 °C; Acetone: Epon 812 = 1:3 for 2–4 h at 37 °C; Pure Epon 812 for 5–8 h at 37 °C. Finally, the samples immersed with pure Epon 812 were cast into the embedding models and then kept at 37 °C oven overnight. The embedding models with resin and samples were moved into 65 °C oven to polymerize for more than 48 h. The resin blocks were sectioned to 60–80 nm thick slices on the ultra-microtome, then captured onto the 150-mesh grids with formvar film. The sections were stained with 2% uranium-acetate-saturated alcohol solution in the dark for 8 min, rinsed in 70% ethanol 3 times, and then rinsed in ultra-pure water 3 times. They were further stained with 2.6% lead citrate for 8 min and then rinsed with ultra-pure water 3 times. Finally, the sections were observed under TEM to record the results.

### 2.7. Statistical Analysis

All experimental data were statistically analyzed using Microsoft Excel 2021 (Microsoft Corporation, Redmond, DC, USA). Graphs were created using Origin 2024 (Origin Lab Corporation, Northampton, MA, USA) and GraphPad Prism 8 (GraphPad Software, Boston, MA, USA). Multiple comparisons were performed using the Bonferroni method in GraphPad Prism 8. GC-IMS data were analyzed, and related figures were generated using the software provided with the instrument (Gesellschaft für Analytische Sensorsysteme mbH, Dortmund Germany).

## 3. Results

### 3.1. Ultrastructural Analysis by TEM

**Figure 1:**
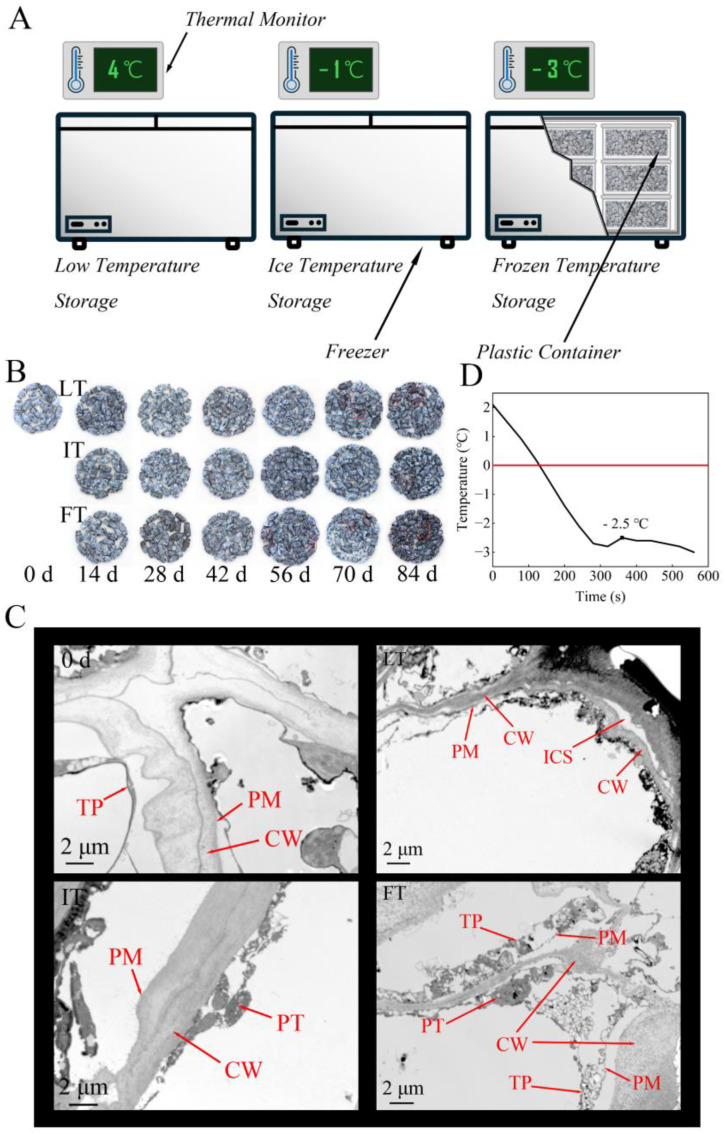
Schematic diagram of storage method (**A**). Photos of fruits during storage (**B**). Transmission electron microscopy. Abbreviations: cell wall (CW), plasma membrane (PM), tonoplast (TP), intercellular spaces (ICS), protoplast (PT) (**C**). Internal temperature changes in blue honeysuckle in freezing-point experiments (**D**).

As depicted in Figure 1B for the appearance of fruits during storage, when no significant change occurred in the IT group up to 84 days, the fruits in the FT treatment showed severe damage in the fruit appearance after 56 days, while those in the LT group began to rot at 70 days. The application of IT storage reduced the morphological variations and decay of the blue honeysuckle. Moreover, the effect of different storage temperatures on the ultrastructure was also investigated using TEM (Figure 1C). At day 0, the cellular structures were clear and easily distinguishable, and the cell walls of adjacent cells were well compact with each other. The plasma membrane adhered tightly to the cell wall, and the tonoplast was smooth and intact. After 84 days of storage, the cell wall structure of LT underwent bending and deformation, creating intercellular spaces between cell walls, while the cytoplasm no longer clung to the cell wall, showing signs of plasmolysis. The double-layer membrane structure of FT showed severe deformation, and the protoplast structure became turbid. The contours of the cell wall became blurred, while the lamella structures could not be discerned. In contrast, the cell wall of the IT group retained its integrity, with the layer morphology well protected. The protoplasts were tightly attached to the cell wall. Collectively, IT storage effectively maintained the intactness of the fruit cells and the integrity of the cell membrane system.

### 3.2. Freezing Point of Blue Honeysuckle

The freezing point of fruits varies depending on their specific structures and composition [28]. The first crucial step in the IT storage is to determine the freezing point of blue honeysuckle. During the determination, the temperature in the fruit core initially dropped to −2.8 °C and subsequently rose to −2.5 °C (Figure 1D). This temperature rebound occurred because the internal tissue of the fruit transited from liquid to solid, simultaneously releasing heat. Consequently, the freezing point of blue honeysuckle was established as −2.5 °C. The interval from 0 °C to −2.5 °C represented the ice temperature storage range. This result aligned with the freezing-point temperatures previously reported for other fruits. For instance, Zhao et al. (2019) [18] measured the freezing point of sweet cherry fruit to be −2.8 °C, while Yang et al. (2021) [28] determined the freezing point of apricot to be −3.0 °C. Given individual differences in fruits and potential fluctuations in the storage environment temperature, the IT was set to −1 °C.

### 3.3. Analysis of Quality Attributes

**Figure 2:**
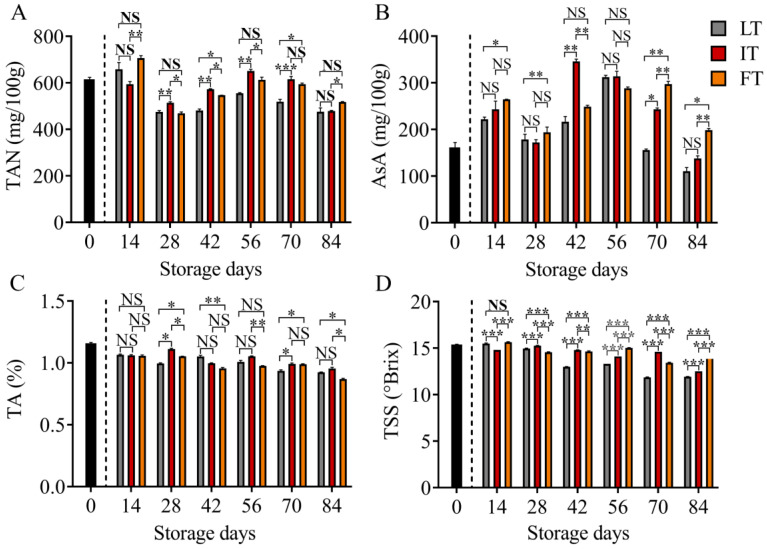
Histogram of quality attributes, namely total anthocyanin content (**A**), ascorbic acid content (**B**), titratable acid content (**C**), and total soluble solid content (**D**). The data were analyzed by multiple comparisons using the Bonferroni method. ***, *p* < 0.001; **, *p* < 0.01; *, *p* < 0.05; NS, no statistical significance.

The flavor of fruits is influenced by various substances, with sugar and acid being crucial factors. Maintaining the content of sugar and acid plays a vital role in preserving the original flavor of fruits [29,30]. IT effectively maintained the content of TSS and TA, enabling the preservation of the original flavor of blue honeysuckle. Figure 2 illustrates the changes in TAN, AsA, TSS, and TA during storage. The TAN content of the IT treatment group was constantly higher than those in other treatments from 28 d to 70 d (Figure 2A). A substantial decrease in TAN was detected in LT. On day 42, when the difference reached the peak, the TAN content of IT was 18.9% higher than LT. Anthocyanin is a significant component of blue honeysuckle [31], possessing excellent antioxidant properties. The effective preservation of anthocyanin content in IT may offer significant health benefits [32,33]. At the beginning of storage, minimal differences in AsA content were observed among all treatments. However, by the end of storage, the AsA content was higher in FT compared to IT, while the content in IT was higher than in LT (Figure 2B). The TSS content in LT consistently exhibited a decreasing trend, while the TSS content in IT and FT remained higher than LT throughout the storage period. The TSS content in IT was significantly higher than other groups on days 28, 42, and 70, while the TSS content in FT was significantly higher than other groups on days 14, 56, and 84. Collectively, these findings suggested that preservation at temperatures below 0 °C favored TSS maintenance, although IT did not demonstrate a significant advantage over FT (Figure 2C). The TA content in all treatments decreased with storage, with IT maintaining the highest content on days 28 and 56–84 (Figure 2D).

### 3.4. Analysis of the VOCs Under Different Storage Temperatures by GC-IMS

#### 3.4.1. Analysis of Topographic Plots

**Figure 3:**
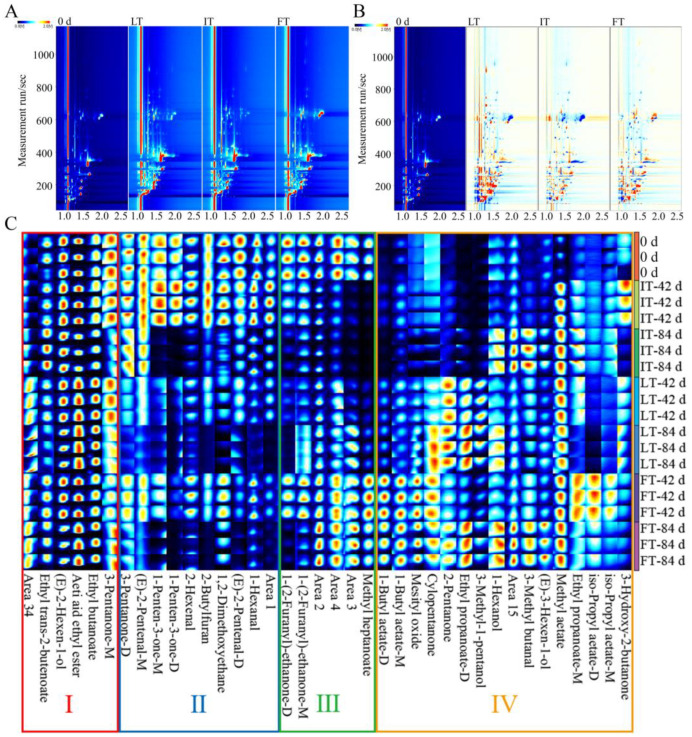
Topographic plots of GC-IMS (**A**). Comparative topographic plots of GC-IMS (**B**). Fingerprint spectrum of GC-IMS on 0 d, 42 d and 84 d (**C**). I: Substances detected in all samples; II: High expression substances in 0 d and IT-42 d; III: High expression substances in 0 d and FT; IV: Substances that do not exist in fresh fruits.

In the topographic plots of Figure 3A, each spot represented a volatile substance. The long red line represented the reactive ion peak generated by the hydrated hydrogen ions in the detector. The detected substance captured the positive charge in the hydrated hydrogen ions, allowing it to move in the migration tube. Due to the different migration times of various substances, they can be distinguished. The leftmost plot was measured at 0 d for fresh fruit, while the three right-hand panels were plots of LT, IT, and FT at 42 d. Due to the loss of commercial value after 84 d of storage, the data at 42 d were used for comparison. In the comparative topographic plots of Figure 3B, the blue background plot on the far left represented the volatile compounds at 0 d, and the three white background plots on the right were comparative topographic plots of LT, IT, and FT on 42 d. If the topographic plots of the group at 42 d and 0 d were the same in a specific area, they had the same concentration. The color was red if the group at 42 d had a higher concentration of a certain VOC than at 0 d. Otherwise, the color was blue. The greater the difference in substance content was, the darker the color was. As observed from the comparative topographic plots, there were many dark blue and red areas in the LT group, indicating that this group lost many original VOCs and produced new compounds. In contrast, the FT group had less composition differences compared to 0 d. The IT group had the least number of red and blue spots among all the treatment groups, suggesting that IT largely retained the original flavor and did not produce unpleasant flavors. Although LT storage is the most common storage method, the fruit quality during storage may rapidly decline [34,35]. This may be attributed to the high level of metabolic activities within the fruit. FT storage slowed down the metabolism by freezing the fruit tissue, but the ice crystals formed during freezing may damage the cellular structure, which can be detrimental to the fruit [35]. The breakage of various plasmalemma in the fruit leads to disruption of the metabolism and direct contact between various substrates and enzymes. Some studies suggested that the formation of ice crystals inside the fruit may also change during the freezing process, thus affecting the internal structure of fruit [36]. IT storage maintained the temperature at the edge of fruit tissue freezing and kept the respiration and metabolism rate low [17], thus allowing the original flavor to be retained. Lipoxygenase-catalyzed unsaturated fatty acids are the main cause of odor [37,38]. IT storage may decrease lipoxygenase activity [18], which may be another way to explain the lesser unfavorable flavor in the IT group. The results of the TEM analysis also demonstrated that the IT group had the best membrane structure integrity, thus maintaining slow and stable metabolism, slowing down fruit flavor deterioration, and making IT the most effective treatment for preserving the original flavor of the fruit.

Through the NIST 2020 RI DB-5 database, 25 volatile substances (monomers and dimers of a substance are counted as one) were identified from the topographic plots (Table 1), including 9 esters, 5 alcohols, 4 ketones, 4 aldehydes, 2 furans, and 1 ether. Esters had the highest percentage of all volatile compounds, up to 36%, while ethers only accounted for 1%. It has been shown that esters are the main VOCs of blue honeysuckle, akin to those reported for other fruits [39,40].

#### 3.4.2. Analysis of Fingerprint Spectrum

The fingerprints in Figure 3C were sorted from top to bottom as follows: 0 d, IT stored for 42 d, IT stored for 84 d, LT stored for 42 d, LT stored for 84 d, FT stored for 42 d, and FT stored for 84 d, with three determinations for each treatment. The detected substances were arranged from left to right for clearer presentation. Regions I, II, and III in Figure 3C include the substances that represent the flavor of fresh blue honeysuckle. All substances in region I (Figure 3C) were present at high levels in all samples, including ethyl butanoate, acetic acid ethyl ester, (E)-2-Hexen-1-ol, and ethyl trans-2-butenoate. The volatiles in regions II and III (Figure 3C) were substances that exhibited differences among the treatment groups. Region II (Figure 3C) contained 1-Hexanal, 1,2-Dimethoxyethane, and 1-Penten-3-one. Region III (Figure 3C) comprise methyl heptanoate, and 1-(2-Furanyl)-ethanone-M. Furans, including 2-Butylfuran in region II (Figure 3C) and 1-(2-Furanyl)-ethanone-M (D) in region III(Figure 3C), are important products of the Maillard reactions in foods and are of interest in many applications, such as coffee and baking flavorings [22,41]. After 42 d of storage, IT demonstrated the best maintenance of the 10 volatiles in region II, while FT was not as effective as IT due to the reduction in volatiles, and LT was the least effective, having lost most of the volatiles. After 84 d of storage, IT still retained (E)-2-Pentenal-M and 3-Pentanone-D, whereas LT and FT lost almost all the volatiles in region II (Figure 3C). Unexpectedly, the FT treatment retained the volatiles in region III (Figure 3C) well. Volatiles in region IV were produced during storage. After 42 d of storage, IT had the least amount of newly produced volatiles, while both LT and FT produced volatiles not found in fresh fruit. After 84 days of storage, eight volatiles were elevated in IT compared to 0 d in region IV (Figure 3C), whereas almost all the volatiles detected in LT and FT were substantially elevated. The production of new odors did not necessarily mean that the flavor may be related to deterioration; on the contrary, the newly produced odors may also be pleasant, such as iso-Propyl acetate, 1-Butyl acetate, and 1-Hexanol, which contribute a fruit flavor, and 3-Hydroxy-2-butanone, which contributes a milk flavor in region IV. Most of the alcohols detected in the experiment were in region IV, which composed significant component of the flavor, giving the fruit an elegant aroma [42]. However, the generation of fewer new odors during storage indicated that the original flavor of the fresh fruit was better preserved. Combined with the results from the previous analyses, it can be concluded that IT has the best impact on maintaining the original VOCs in blue honeysuckles.

In summary, IT storage can be applied to the postharvest handling and storage of fresh blue honeysuckle to retain the original flavor of the fruits, while FT storage has shown that blue honeysuckle can be used for flavoring by adding it to cold drinks such as ice cream.

#### 3.4.3. Principal Component Analysis

**Figure 4:**
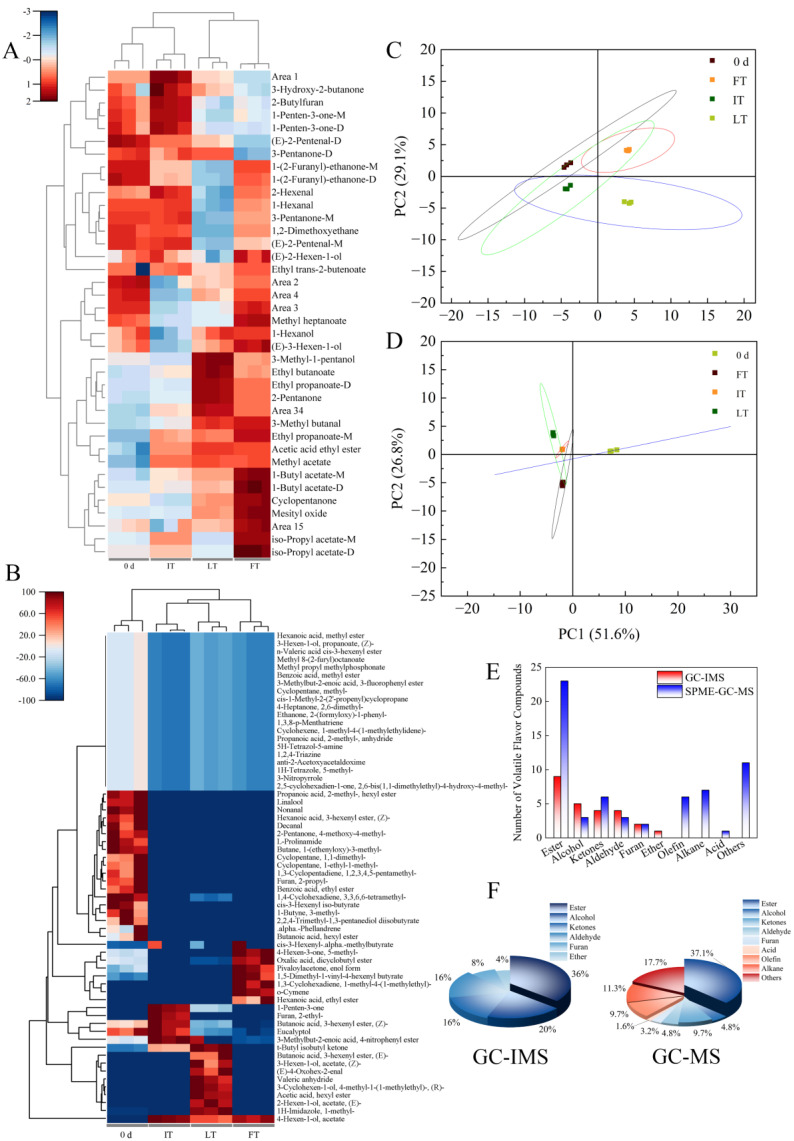
Heatmap of VOCs in GC-IMS (**A**). Heatmap of VOCs in GC-MS (**B**). The principal component analysis (PCA) of VOCs in GC-IMS (**C**). The PCA of VOCs in GC-IMS (**D**). Composition of substances detected besides GC-IMS and SPME-GC-MS (**E**,**F**).

Principal component analysis (PCA) is a multivariate statistical analysis that can be applied to define a few principal component factors representing many complex variables in an experiment [43]. Two-dimensional planar analysis was employed to identify patterns such as similarities and differences observed in different treatments. In Figure 4C, PC 1 contributed 49.7%, and PC 2 contributed 29.1%, resulting in a cumulative contribution of 78.8%, which elucidated the differences and similarities between treatments. A significant difference between different treatments at 42 d was obtained, with IT exhibiting the highest similarity to fruit volatiles at 0 d. The heatmap in Figure 3A revealed a high similarity in the production of volatiles between the IT and 0 d controls. Similar conclusions can be drawn from the cluster analysis, in which IT and 0 d were grouped together.

### 3.5. Analysis of the VOCs Under Different Storage Temperatures by GC-MS

The VOCs of blue honeysuckle were analyzed using SPME-GC-MS. The SPME method, an efficient technique for extracting VOCs, was employed to extract the volatiles [44]. A total of 62 volatiles were detected in blue honeysuckle via GC-MS, including 23 esters, 7 alkanes, 6 ketones, 6 olefins, 3 aldehydes, 3 alcohols, 2 furans, 1 acid, and 11 others (Table 2). The key volatiles identified in blue honeysuckle were Hexanoic acid, ethyl ester, Decanal, Nonanal, Eucalyptol, and Linalool, which was consistent with the findings of Xia et al. (2023) [13]. The PCA in Figure 4D demonstrated that IT storage closely resembled the performance at 0 d, with PC 1 contributing 51.6%, PC 2 contributing 26.8%, and a total of 78.4%, indicating a strong correlation. The heatmap in Figure 4B illustrates the substantial differences among various treatments and 0 d.

### 3.6. Comparative Analysis of GC-IMS and GC-MS

In this study, 25 substances were detected using GC-IMS, while 62 substances were identified with GC-MS. As shown in Figure 4E, both methods detected esters, alcohols, ketones, aldehydes, and furans. Additionally, GC-IMS detected ethers, whereas GC-MS identified olefins, aluminum, acids, and various other substances. The compounds detected by GC-MS were more comprehensive, while GC-IMS exhibited high sensitivity to specific substances. The differences in the VOCs characterized by the two methods can be attributed to several factors: 1. Different pre-treatment; 2. different extraction methods for aroma compounds; 3. different detection principles of the instruments; and 4. variations in the spectrum libraries of the processing software. As illustrated in Figure 4F, esters accounted for the largest proportion of detected substances in both methods.

GC-IMS detection involved the initial separation of substances through the chromatographic column, followed by their introduction into the IMS tube, where the particles were distinguished based on their different drift rates in the electric field [45]. A unique characteristic of GC-IMS is its ability to differentiate between monomers and polymers. IMS operates at atmospheric pressure, eliminating the need for instrument vacuum time. However, during the analysis process, ion–ion and ion–molecule competition reactions occur, which can reduce the substance discrimination capability of IMS [46]. Although GC-IMS effectively demonstrates differences and changes between samples, accurate quantification remains challenging. When employing GC-MS, the SPME method is necessary for extraction to achieve optimal detection results, which is time-consuming and complex to perform. Nevertheless, GC-MS enables accurate characterization through ion fragment comparison and is suitable for quantitative determination. This method also exhibits strong separation and recognition capabilities [47]. Both GC-IMS and GC-MS have their advantages and limitations. They can be used in combination or selected based on specific experimental requirements. In practical applications, GC-IMS is commonly used for rapid detection scenarios, such as distinguishing the quality of edible oil, the type and quality of coffee beans, etc. By contrast, GC-MS is often used for component determination in professional fields due to its comprehensive spectral library and accurate detection capabilities, which is commonly used in fields such as medicine, biology, and chemistry [48,49,50].

## 4. Conclusions

In summary, IT storage is the most effective method for maintaining volatile compounds in blue honeysuckle under the present experimental conditions. GC-IMS detected 9 esters, 5 alcohols, 4 ketones, 4 aldehydes, 2 furans, and 1 ether, while GC-MS identified 23 esters, 7 alkanes, 6 ketones, 6 olefins, 3 aldehydes, 3 alcohols, 2 furans, 1 acid, and 11 other compounds. During IT storage, the internal fruit tissues were damaged the least, while the contents of TSS, TA, and total anthocyanins were well preserved, ensuring the postharvest quality of fruit. In conclusion, IT storage is a suitable method for blue honeysuckle storage, particularly for retaining volatile flavor compounds. This study substantially contributes to the development of blue honeysuckle industry for fresh food sales and expands the application of GC-IMS in fruit storage experiments. Notably, the present study did not address which metabolic pathways were specifically affected by temperature changes, but this deserves further efforts. In the future, it would be appealing to investigate whether ice-temperature storage combined with other methods can more effectively prolong the storage time of blue honeysuckle, while applications in other berry fruits and examinations of influencing environmental factors may become new research directions.

## Figures and Tables

**Table 1:** Volatile compounds identified by GC-IMS.

Category	No.	Compound	CAS#	MW	RI	RT [sec]	Dt [a.u.]	Aroma Characteristics
Esters (9)	1	Acetic acid ethyl ester	141-78-6	88.1	614.8	148.578	1.33298	Fruity
	2	Methyl acetate	79-20-9	74.1	540.2	120.785	1.18873	Special aroma
	3	Ethyl butanoate	105-54-4	116.2	790.6	268.747	1.55056	Fresh aroma, Fruity
	4	Methyl heptanoate	106-73-0	144.2	1020	615.058	1.80562	Berry, Iris, Fruity
	5	Mesityl oxide	141-79-7	98.1	784.7	263.245	1.44037	Fruity
	6	1-Butyl acetate-M	123-86-4	116.2	805.8	283.67	1.2346	Fruity
		1-Butyl acetate-D	123-86-4	116.2	805.8	283.67	1.2346	Fruity
	7	Ethyl propanoate-M	105-37-3	102.1	708.7	196.933	1.15528	Pineapple
		Ethyl propanoate-D	105-37-3	102.1	708.7	196.933	1.15528	Pineapple
	8	iso-Propyl acetate-M	108-21-4	102.1	655.9	166.56	1.15934	Special fruity aroma
		iso-Propyl acetate-D	108-21-4	102.1	655.9	166.56	1.15934	Special fruity aroma
	9	Ethyl trans-2-butenoate	623-70-1	114.1	838.9	318.841	1.1776	Spicy, Rum, Jackfruit
Alcohols (5)	10	1-Hexanol	111-27-3	102.2	873.5	360.257	1.63716	Fruity
	11	3-Hydroxy-2-butanone	513-86-0	88.1	737.7	220.007	1.35493	Milk
	12	3-Methyl-1-pentanol	589-35-5	102.2	841.6	321.92	1.59607	Spicy, Cocoa, Wine
	13	(E)-3-Hexen-1-ol	928-97-2	100.2	859.9	343.432	1.24638	Grass
	14	(E)-2-Hexen-1-ol	928-95-0	100.2	853.9	336.164	1.51193	Pleasant odor
Ketones (4)	15	Cyclopentanone	120-92-3	84.1	783	261.57	1.32827	Mint
	16	2-Pentanone	107-87-9	86.1	700.5	190.91	1.39642	Spicy, Fruity
	17	3-Pentanone-M	96-22-0	86.1	695.7	187.418	1.11127	-
		3-Pentanone-D	96-22-0	86.1	695.7	187.418	1.11127	-
	18	1-Penten-3-one-D	1629-58-9	84.1	682.8	179.511	1.30614	Tangerine, Onion
		1-Penten-3-one-M	1629-58-9	84.1	682.8	179.511	1.30614	Tangerine, Onion
Aldehydes (4)	19	1-Hexanal	66-25-1	100.2	798.2	276.055	1.28327	Fruity, Vegetable aroma
	20	2-Hexenal	505-57-7	98.1	858.9	342.224	1.17677	Leaf aroma
	21	3-Methyl butanal	590-86-3	86.1	654.6	165.965	1.40331	Chocolate, Cocoa, Fruity (diluted)
	22	(E)-2-Pentenal-M	1576-87-0	84.1	749.3	230.009	1.10366	-
		(E)-2-Pentenal-D	1576-87-0	84.1	749.3	230.009	1.10366	-
Furans (2)	23	2-Butylfuran	4466-24-4	124.2	893.4	386.729	1.17853	-
	24	1-(2-Furanyl)-ethanone-M	1192-62-7	110.1	912.1	415.535	1.12334	Fruity, Sweet
		1-(2-Furanyl)-ethanone-D	1192-62-7	110.1	912.1	415.535	1.12334	Fruity, Sweet
Ether (1)	25	1,2-Dimethoxyethane	110-71-4	90.1	635.4	157.328	1.09696	Pungent odor, Sweet

**Table 2:** Volatile compounds identified by SPME-GC-MS.

Category	NO.	Compound	CAS	Concentration (ng/g)						
				0 d	42 d			84 d		
				CK	FT	IT	LT	FT	IT	LT
Esters (23)	1	Hexanoic acid, methyl ester	106-70-7	-	-	-	-	-	0.64±0.05	-
	2	Hexanoic acid, ethyl ester	123-66-0	2.02 ± 0.06	-	7.73 ± 0.13	12.42 ± 0.38	54.98 ± 1.89	1.14 ± 0.31	47.78 ± 4.47
	3	Acetic acid, hexyl ester	142-92-7	28.11 ± 0.81	286.26 ± 0.00	219.94 ± 0.00	79.92 ± 0.00	815.90 ± 21.07	26.11 ± 4.85	91.57 ± 7.18
	4	Propanoic acid, 2-methyl-, hexyl ester	2349-07-7	2.19 ± 0.16	-	-	-	-	0.83 ± 0.11	-
	5	2-Hexen-1-ol, acetate, (E)-	2497-18-9	23.64 ± 0.80	185.20 ± 7.58	195.76 ± 4.78	157.42 ± 2.69	367.94 ± 17.46	6.07 ± 0.88	27.28 ± 3.26
	6	Butanoic acid, hexyl ester	2639-63-6	1.95 ± 0.08	4.56 ± 0.20	-	-	-	-	-
	7	3-Hexen-1-ol, propanoate, (Z)-	33467-74-2	-	-	-	-	7.53 ± 1.19	-	-
	8	n-Valeric acid cis-3-hexenyl ester	35852-46-1	-	-	-	-	-	1.20 ± 0.44	-
	9	3-Hexen-1-ol, acetate, (Z)-	3681-71-8	70.33 ± 2.51	-	-	-	-	-	-
	10	Methyl 8-(2-furyl)-octanoate	38199-50-7	-	-	-	-	-	4.17 ± 0.93	-
	11	cis-3-Hexenyl iso-butyrate	41519-23-7	6.31 ± 0.83	-	-	-	-	-	-
	12	Butanoic acid, 3-hexenyl ester, (E)-	53398-84-8	17.30 ± 1.16	37.22 ± 2.60	-	2.76 ± 0.45	78.20 ± 3.18	2.93 ± 0.61	-
	13	cis-3-Hexenyl-alpha-methylbutyrate	53398-85-9	1.07 ± 0.20	5.08 ± 0.73	-	-	24.95 ± 1.87	-	-
	14	Methyl propyl methylphosphonate	683-25-0	-	-	-	-	-	-	564.37 ± 14.33
	15	2,2,4-Trimethyl-1,3-pentanediol diisobutyrate	6846-50-0	3.04 ± 0.25	-	-	-	9.24 ± 0.75	0.87 ± 0.10	-
	16	4-Hexen-1-ol, acetate	72237-36-6	-	-	415.24 ± 17.20	154.24 ± 5.87	-	21.35 ± 2.16	83.12 ± 3.19
	17	1,5-Dimethyl-1-vinyl-4-hexenyl butyrate	78-36-4	-	-	-	4.84 ± 0.98	-	-	-
	18	Benzoic acid, methyl ester	93-58-3	-	-	-	-	-	1.44 ± 0.22	-
	19	Benzoic acid, ethyl ester	93-89-0	-	-	-	2.85 ± 0.26	-	-	-
	20	3-Methylbut-2-enoic acid, 4-nitrophenyl ester	1000307-59-8	-	-	-	28.38 ± 1.89	90.81 ± 7.19	-	-
	21	Oxalic acid, dicyclobutyl ester	1000309-69-5	-	4.60 ± 1.30	-	-	-	-	-
	22	3-Methylbut-2-enoic acid, 3-fluorophenyl ester	1000331-15-0	-	-	-	-	-	5.68 ± 0.39	-
	23	Hexanoic acid, 3-hexenyl ester, (Z)-	31501-11-8	1.00 ± 0.23	-	-	-	-	-	-
Alkanes (7)	24	Cyclopentane, methyl-	96-37-7	-	-	-	-	78.50 ± 4.51	-	-
	25	2-Pentanone, 4-methoxy-4-methyl-	107-70-0	0.87 ± 0.21	-	-	-	-	-	-
	26	Cyclopentane, 1,1-dimethyl-	1638-26-2	1.14 ± 0.18	-	-	-	-	-	-
	27	Butanoic acid, 3-hexenyl ester, (Z)-	16491-36-4	-	-	20.36 ± 1.34	-	-	-	-
	28	Cyclopentane, 1-ethyl-1-methyl-	16747-50-5	1.23 ± 0.61	-	-	-	-	-	-
	29	Butane, 1-(ethenyloxy)-3-methyl-	39782-38-2	0.85 ± 0.10	-	-	-	-	-	-
	30	cis-1-Methyl-2-(2′-propenyl)-cyclopropane	76588-97-1	-	-	-	-	491.30 ± 26.82	-	-
Ketones (6)	31	4-Heptanone, 2,6-dimethyl-	108-83-8	-	-	-	-	-	9.86 ± 1.37	-
	32	4-Hexen-3-one, 5-methyl-	13905-10-7	1.34 ± 0.29	3.43 ± 0.41	-	-	-	-	-
	33	t-Butyl isobutyl ketone	14705-50-1	-	-	-	20.06 ± 1.35	-	-	-
	34	1-Penten-3-one	1629-58-9	17.83 ± 2.30	-	-	-	-	-	-
	35	Ethanone, 2-(formyloxy)-1-phenyl-	55153-12-3	-	-	-	-	-	4.23 ± 0.32	-
	36	Pivaloylacetone, enol form	1000202-24-3	-	6.44 ± 0.57	-	-	-	-	-
Olefins (6)	37	1,3,8-p-Menthatriene	18368-95-1	-	-	-	-	-	-	155.85 ± 12.49
	38	1,4-Cyclohexadiene, 3,3,6,6-tetramethyl-	2223-54-3	-	-	-	1.44 ± 0.28	-	-	-
	39	1,3-Cyclopentadiene, 1,2,3,4,5-pentamethyl-	4045-44-7	1.59 ± 0.34	-	-	-	-	-	-
	40	Cyclohexene, 1-methyl-4-(1-methylethylidene)-	586-62-9	-	-	-	-	-	-	27.94 ± 2.98
	41	Alpha-Phellandrene	99-83-2	3.32 ± 0.66	-	-	-	-	-	-
	42	1,3-Cyclohexadiene, 1-methyl-4-(1-methylethyl)-	99-86-5	-	-	-	8.76 ± 1.21	-	-	86.24 ± 5.25
Aldehydes (3)	43	Decanal	112-31-2	0.91 ± 0.19	-	-	-	-	-	-
	44	Nonanal	124-19-6	1.84 ± 0.51	-	-	-	-	-	-
	45	(E)-4-Oxohex-2-enal	1000374-04-2	62.05 ± 3.31	10.34 ± 1.08	99.42 ± 4.67	37.52 ± 2.16	21.04 ± 1.59	26.78 ± 1.47	-
Alcohols (3)	46	3-Cyclohexen-1-ol, 4-methyl-1-(1-methylethyl)-, (R)-	20126-76-5	3.35 ± 0.11	-	-	147.08 ± 5.06	-	-	-
	47	Eucalyptol	470-82-6	13.63 ± 1.49	4.42 ± 0.38	14.62 ± 1.30	6.71 ± 0.46	9.20 ± 0.89	27.53 ± 3.09	-
	48	Linalool	78-70-6	2.35 ± 0.14	-	-	-	26.74 ± 1.15	2.09 ± 0.33	-
Furan (2)	49	Furan, 2-ethyl-	3208-16-0	16.67 ± 1.03	-	-	2.33 ± 0.35	-	3.50 ± 0.69	-
	50	Furan, 2-propyl-	4229-91-8	1.48 ± 0.60	-	-	-	-	-	-
Acid (1)	51	Propanoic acid, 2-methyl-, anhydride	97-72-3	-	-	-	-	-	24.51 ± 0.95	-
Others (11)	52	5H-Tetrazol-5-amine	1000273-02-0	-	-	-	-	-	-	38.84 ± 1.15
	53	Valeric anhydride	2082-59-9	27.30 ± 1.82	4.40 ± 0.50	57.17 ± 1.25	53.14 ± 0.00	45.03 ± 2.57	0.87 ± 0.32	-
	54	1,2,4-Triazine	290-38-0	-	-	-	-	24.38 ± 2.13	-	-
	55	anti-2-Acetoxyacetaldoxime	37858-07-4	-	-	-	-	17.94 ± 3.48	-	-
	56	1H-Tetrazole, 5-methyl-	4076-36-2	-	-	-	-	85.14 ± 0.52	-	-
	57	3-Nitropyrrole	5930-94-9	-	-	-	-	26.43 ± 0.78	-	-
	58	1-Butyne, 3-methyl-	598-23-2	5.18 ± 0.83	-	-	-	-	-	-
	59	1H-Imidazole, 1-methyl-	616-47-7	-	281.83 ± 16.97	-	-	-	-	-
	60	L-Prolinamide	7531-52-4	0.83 ± 0.13	-	-	-	-	-	-
	61	2,5-cyclohexadien-1-one, 2,6-bis(1,1-dimethylethyl)-4-hydroxy-4-methyl-	1000401-12-0	-	-	-	-	16.74 ± 1.04	-	-
	62	o-Cymene	527-84-4	-	-	-	8.69 ± 0.46	-	-	-

## Data Availability

The original contributions presented in the study are included in the article, further inquiries can be directed to the corresponding authors.
